# BiP Negatively Affects Ricin Transport

**DOI:** 10.3390/toxins5050969

**Published:** 2013-05-10

**Authors:** Tone F. Gregers, Sigrid S. Skånland, Sébastien Wälchli, Oddmund Bakke, Kirsten Sandvig

**Affiliations:** 1 Department of Biosciences, and Centre for Immune Regulation, University of Oslo, Oslo 0316, Norway; E-Mails: t.f.gregers@ibv.uio.no (T.F.G.); sigrid.skanland@ncmm.uio.no (S.S.S.); oddmund.bakke@ibv.uio.no (O.B.); 2 Section of Biochemistry, Institute for Cancer Research, Oslo University Hospital Radiumhospitalet, Oslo 0379, Norway; E-Mail: Sebastien.Walchli@rr-research.no; 3 Section of Immunology, Institute for Cancer Research, Oslo University Hospital Radiumhospitalet, Oslo 0379, Norway; 4 Centre for Cancer Biomedicine, Faculty of Medicine, University of Oslo, Oslo 0379, Norway

**Keywords:** BiP, ricin, toxin, ER chaperones, endoplasmic reticulum, toxicity

## Abstract

The AB plant toxin ricin binds both glycoproteins and glycolipids at the cell surface *via* its B subunit. After binding, ricin is endocytosed and then transported retrogradely through the Golgi to the endoplasmic reticulum (ER). In the ER, the A subunit is retrotranslocated to the cytosol in a chaperone-dependent process, which is not fully explored. Recently two separate siRNA screens have demonstrated that ER chaperones have implications for ricin toxicity. ER associated degradation (ERAD) involves translocation of misfolded proteins from ER to cytosol and it is conceivable that protein toxins exploit this pathway. The ER chaperone BiP is an important ER regulator and has been implicated in toxicity mediated by cholera and Shiga toxin. In this study, we have investigated the role of BiP in ricin translocation to the cytosol. We first show that overexpression of BiP inhibited ricin translocation and protected cells against the toxin. Furthermore, shRNA-mediated depletion of BiP enhanced toxin translocation resulting in increased cytotoxicity. BiP-dependent inhibition of ricin toxicity was independent of ER stress. Our findings suggest that in contrast to what was shown with the Shiga toxin, the presence of BiP does not facilitate, but rather inhibits the entry of ricin into the cytosol.

## 1. Introduction

Folding and assembly of secretory and transmembrane proteins occur in the endoplasmic reticulum (ER) and are assisted by numerous molecular chaperones. The two principal chaperone systems in the ER are calnexin/calreticulin and BiP/Grp94 [1]. Unfolded or misfolded proteins that accumulate in the ER must either be refolded or degraded to maintain the homeostasis of the ER. Degradation of ER substrates is achieved by retrotranslocation through the Sec61 translocon to the cytosol, ubiquitinylation and degradation by the proteasome. This process is called ER associated degradation (ERAD).

BiP, also known as Grp78, belongs to the Hsp70 family and was the first ER chaperone to be identified [2,3]. BiP is an ATPase that binds transiently to newly synthesized proteins translocated into the ER, and for a longer period of time to underglycosylated, misfolded, or unassembled proteins. BiP has been described as the master regulator of ER function, being involved not only in folding and assembly of newly synthesized proteins, but also in maintaining the permeability barrier of the ER during protein translocation, targeting misfolded proteins for retrograde translocation and sensing conditions of ER-stress, to activate the mammalian unfolded protein response (reviewed in [4]). 

Ricin is an AB protein toxin found in the seeds of the plant *Ricinus communis*. It consists of two subunits, A and B chain, held together by a disulfide bridge. The B-chain has a lectin function and binds to glycoproteins and glycolipids on the cell surface. Ricin is endocytosed by both clathrin-dependent and -independent mechanisms and then sorted to the Golgi apparatus (reviewed in [5]). Ricin is finally transported to the ER, where the disulfide bond connecting the A- and B-chain has been shown to be reduced by the protein disulfide isomerase (PDI) and thioredoxin reductase [6,7]. It was proposed that this reduction partially unfolds the A-chain, enabling this subunit to cross the ER membrane (reviewed in [8]).The A-chain refolds upon arrival into the cytosol which enables it to inactivate the ribosomes, thereby exerting its toxic effect. Cytosolic chaperones Hsc70 and Hsc90 were found to be essential for refolding of ricin A into a functional toxic enzyme [9]. Furthermore, in yeast, the BiP orthologue Kar2p was found to be critical for maximum toxicity [10]. Within the ER, ricin was shown to interact with Sec61 [11] and the ER degradation enhancing α-mannosidase I like protein (EDEM) [12,13], which indicates that ricin, like other protein toxins, exploits the ERAD pathway to access the cytosol [14]. Recently two separate siRNA screens were performed and provided a list of proteins involved in ricin transport from the plasma membrane to the cytosol, including ER residents [15,16]. Only Moreau and colleagues tested BiP knockdown; they reported no inhibitory effect, but did not test for enhancement of toxicity [15,16]. Indeed, BiP has been implicated in transport of other toxins to the cytosol. The B-chain of the bacterial AB_5_ toxin Shiga was shown to bind both BiP [17] and the BiP co-chaperone HEDJ/ERdj3 [18]. In addition, transport of cholera toxin from ER-derived microsomes is both BiP- [19] and PDI-dependent [20,21]. BiP is thought to inhibit aggregation of cholera toxin in the ER and thus makes it export-competent after reduction [19]. Furthermore, it has recently been published that the A_2_B toxin Anthrax depends on BiP for proper retrotranslocation [22]. 

In the present study, we have investigated the role of BiP in ricin toxicity, and based on our results, we propose that BiP, in contrast to its importance for retro-translocation of other toxins studied so far, acts as a negative regulator of ricin toxicity.

## 2. Results

### 2.1. BiP Protects against Ricin Toxicity

**Figure 1:**
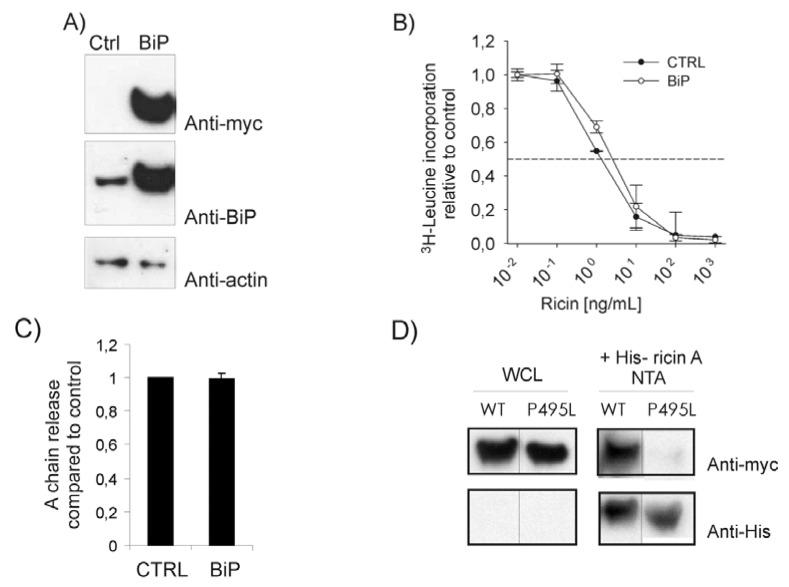
(**A**) HEK293 cells were transfected with BiP or an empty vector (ctrl). Lysates were run on SDS-PAGE, transferred to a PVDF membrane and examined for BiP expression using an anti-myc antibody or anti-BiP antibody. Anti-actin was used for loading control. (**B**) Cells were transfected as indicated and incubated with increasing amounts of ricin in leucine-free medium for 3 h, then washed and incubated with 1 µCi/mL [^3^H]leucine for 20 min. The incorporation of [^3^H]leucine was measured and data presented relative to the control (CTRL). This experiment was repeated three times with similar results in duplicates. (**C**) Cells transfected as indicated were pre-incubated with 0.2 mCi/mL ^35^SO_4_^2-^ for 3 h and then with ricin sulf-1 for an additional 3 h. The cells were lysed and ricin was immunoprecipitated and subjected to SDS-PAGE under non-reducing conditions. The amount of ricin A-chain reduced from the holotoxin in each sample was quantified and compared to total sulfated ricin in the samples. (**D**) Cells were transfected with myc-tagged BiP constructs as indicated (wild type, WT or substrate binding mutant, P495L) before lysis and incubated with 1 μg His-tagged ricin A-chain for 1 h. The toxin was pulled down using a Ni-NTA column. The beads were separated in a SDS-PAGE and analyzed by Western blot. BiP proteins were detected using anti-myc antibodies and ricin with anti-His antibodies. Whole cell lysates (WCL, left panels) and pull down (+His-ricin A NTA, right panels) are shown.

In order to investigate the role of BiP in ricin toxicity, HEK293 cells were first transiently transfected with myc-tagged wild type BiP (Figure 1A and [23]). Over-expression of BiP resulted in a small but significant protection against ricin toxicity compared to cells transfected with a control vector (Figure 1B). The IC_50_ was increased with a factor of 1.4 ± 0.03 (standard error), *p* < 0.005. These data indicate that BiP plays a role in ricin toxicity.

It is accepted that ricin holotoxin can be reduced in the ER prior to translocation, and that mainly the A-chain is translocated to the cytosol. Both PDI and thioredoxin reductase have been implicated in the disulfide bond reduction of ricin in the ER [6,7]. Protection against ricin in BiP transfected cells could therefore be due to impaired reduction of the toxin in the ER. To investigate this, BiP-transfected cells were incubated with ricin sulf-1 [24], a modified ricin molecule containing a sulfation site in the A-chain, in the presence of ^35^SO_4_^2-^ containing medium, in order to radiolabel the A-chain. As shown in Figure 1C, over-expression of BiP did not alter the A-chain release after three hours of ricin incubation, indicating that reduction of holotoxin was not impaired. Similar data were obtained after 90 min of ricin incubation (data not shown), suggesting that over-expression of BiP does not affect reduction of ricin holotoxin.

Finally, we tested the ability of ricin to bind to BiP. HEK293 cells were transiently transfected with constructs encoding myc-tagged BiP WT or a mutant with a reduced substrate affinity, namely BiP P495L [23,25] and lysates were prepared. We constructed an active His-tagged ricin A-chain (data not shown) and performed pull down assays. Purified toxin A-chain was added to the lysate and Ni-NTA column was used to pull down His-ricin A. The presence of BiP in the precipitate was determined by Western blot using anti-myc antibodies. As shown in Figure 1D, BiP WT was found to interact with His-ricin A, whereas the binding mutant was only detected as residual signals, suggesting that ricin A-chain is dependent on a functional substrate binding site on BiP. However, it cannot be excluded that additional interaction partners were required. 

### 2.2. Depletion of BiP Sensitizes Cells towards Ricin Toxicity

**Figure 2:**
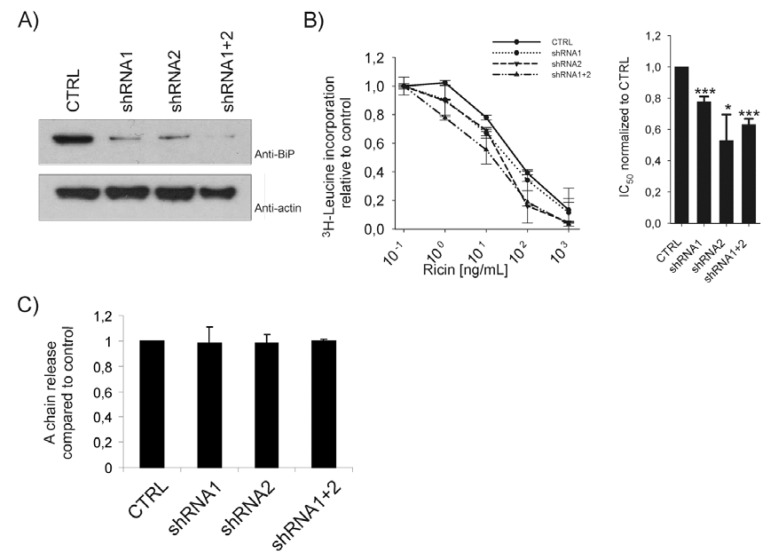
(**A**) Cells were transfected with a control shRNA vector or two different BiP shRNA constructs either alone or in combination, for three days. Cell lysates were analysed by Western blot using anti-BiP and anti-actin antibodies. (**B**) Cells transfected with the indicated shRNA were incubated with increasing amounts of ricin in leucine-free medium for 3 h, then washed and incubated with 1 µCi/mL [^3^H]leucine for 20 min. The incorporation of [^3^H]leucine was measured and data presented relative to the control (CTRL). Left panel: a representative experiment performed with parallels. Right panel: average IC_50_ values of 3–4 experiments. Error bars represent standard error of the mean (*p* < 0.001 for shRNA1 (*n* = 4) and shRNA1+2 (*n* = 3) and *p* = 0.047 for shRNA2 (*n* = 3)). (**C**) Cells transfected with the indicated shRNA constructs were incubated with 0.2 mCi/mL^35^SO_4_^2-^ for 3 h and then with ricin sulf-1 for an additional 3 h. The cells were lysed and ricin was immunoprecipitated and subjected to SDS-PAGE under non-reducing conditions. The amount of ricin A-chain in each sample was quantified and compared to total sulfated ricin in the samples.

To further investigate the role of BiP in ricin toxicity, we used short hairpin RNA (shRNA) to knock down BiP. HEK293 cells were transfected with two different shRNA constructs targeted against different regions of human BiP mRNA. Both constructs efficiently knocked down endogenous BiP (Figure 2A). Knockdown of co-transfected myc-tagged BiP wt was as efficient as knockdown of endogenous BiP (data not shown). Toxicity experiments performed on cells transfected with BiP shRNAs showed a clear sensitization towards ricin (Figure 2B, left panel). The IC_50_ values from two different experiments performed with parallels were calculated and showed a sensitizing effect of BiP depletion relative to control (Figure 2B, right panel). This sensitization was not due to altered reduction of ricin, since the A-chain released from the holotoxin was found to be unaffected in cells transfected with BiP shRNA constructs (Figure 2C). This confirms that BiP has no direct role in di-sulfide bond reduction of ricin, although BiP is clearly involved in ricin toxicity. One possible explanation for the protection observed upon BiP overexpression and the sensitization observed upon BiP depletion is that ricin might be retained in the ER by BiP. 

### 2.3. The Protein Level of BiP Affects Ricin Translocation to the Cytosol

**Figure 3:**
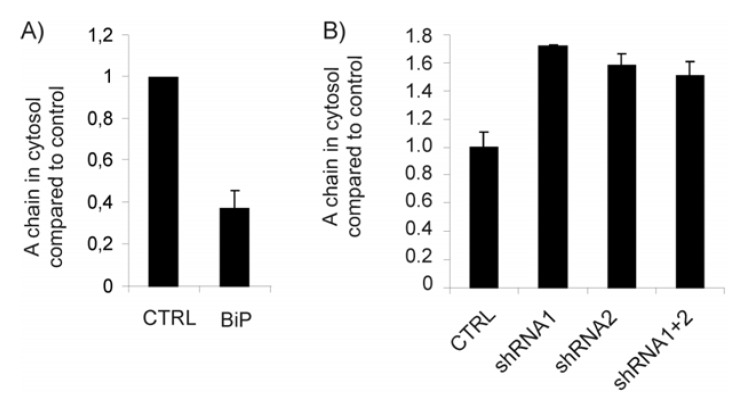
(**A** and **B**) Cells transfected as indicated were incubated with 0.2 mCi/mL ^35^SO_4_^2-^ for 3 h and then with ricin sulf-1 for an additional 3 h. The cells were then permeabilized in 3 µg/mL digitonin before lysis and separation of the cytosolic/membrane fractions. Ricin was then immunoprecipitated from each fraction and subjected to SDS-PAGE under reducing conditions. The amount of ricin A-chain in the cytosol was quantified and compared to total sulfated ricin in the samples.

Ricin needs to access the cytosol in order to exert its cytotoxic effect [9]. To further investigate the role of BiP in ricin translocation, we performed a sulfation-permeabilization experiment where translocation of sulfated ricin A-chain from ER to the cytosol was measured [12]. HEK293 cells were transfected with BiP or an empty vector and after 24 h the cells were labeled as for the sulfation experiment (see Materials and Methods). Prior to lysis, the cells were treated with 3 µg/mL digitonin for 30 min. Digitonin is a cholesterol binding glycoside and was used to permeabilize the plasma membrane in order to separate the cytosolic fraction from the ER. Ricin was then immunoprecipitated from each fraction and subjected to SDS-PAGE. Figure 3A shows a quantification of ricin A-chain in the cytosol that was reduced by more than 60% in cells overexpressing BiP. Thus, these results suggest that ricin is retained within the ER when BiP is overexpressed and are in agreement with the reduced toxicity previously reported. 

A similar sulfation-permeabilization experiment was then performed on cells transfected with BiP shRNA. In agreement with the toxicity data, BiP depletion clearly increased the amount of A-chain in the cytosol (Figure 3B), suggesting that ricin retention is counteracted when BiP is depleted. Taken together, our data suggest that BiP inhibits translocation of ricin A-chain to the cytosol.

### 2.4. ER Stress Does Not Cause Increased Ricin Toxicity *per se*

**Figure 4:**
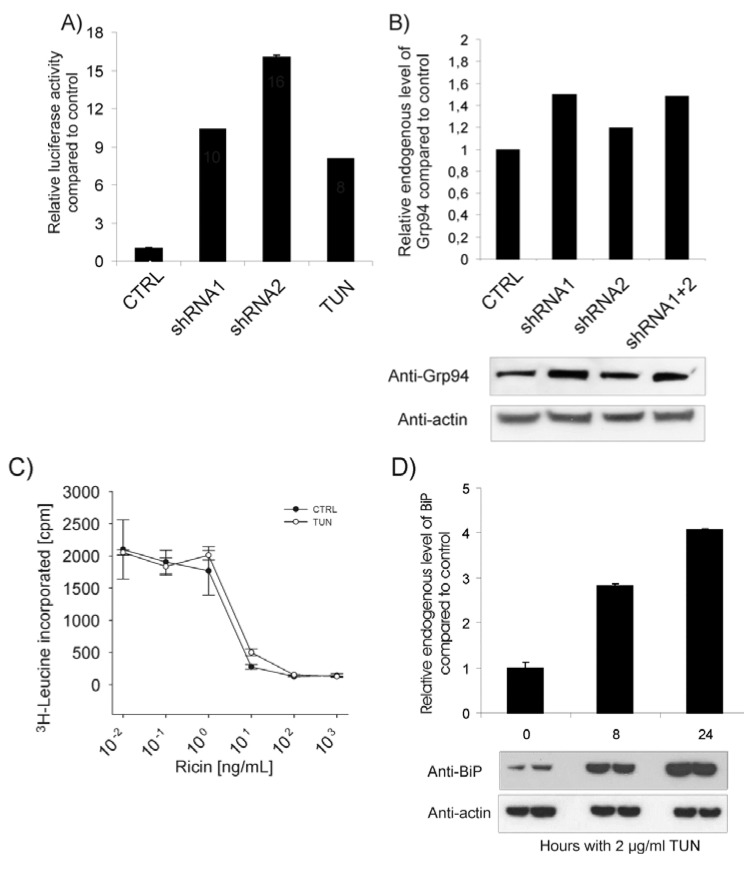
(**A**) Cells were transfected with control vector or BiP shRNA constructs together with an ATF6-luciferase reporter construct and *Renilla* luciferase. 2 μg/mL tunicamycin was used as positive control. Cells were analyzed for luciferase activity 3 days post transfection and the relative activity was normalized to the *Renilla* luciferase internal control. (**B**) Lysate from cells transfected with the shRNA constructs were analyzed for the level of Grp94 by Western blot using rat anti-Grp94. The intensity of each band was quantified by ImageQuant 5.0 software. (**C**) Non transfected cells were incubated with leucine-free medium with or without 2 µg/mL tunicamycin (TUN) for 21 h before different concentrations of ricin were added for subsequently 3 h, then washed in leucine-free medium and incubated with 1 µCi/mL [^3^H]leucine for 20 min. The amount of radioactive protein was finally measured. (**D**) Cells were treated with 2 µg/mL tunicamycin (TUN) for 0, 8 and 24 h in parallels before lysis. Lysates were subjected to SDS-PAGE and Western blot analysis. Anti-BiP was used to detect BiP and anti-actin was used as loading control. The intensity of each band was quantified by ImageQuant 5.0 software where endogenous level of BiP at time point 0 was used as the reference level.

BiP is important for the ER function and known to regulate the activation of the unfolded protein response (UPR, ER stress response). BiP binds to and regulates the activity of ATF6, a transcription factor activated by ER stress conditions [26,27,28]. Upon BiP release, ATF6 is transported to the Golgi apparatus where it is subject to a regulated intramembrane proteolysis. This leads to liberation of the ATF6 cytosolic domain, which in turn triggers its activation as a transcription factor. We therefore suspected that removal of BiP could initiate an ER-stress signaling cascade through ATF6 [29,30], and thus cause the sensitization toward ricin that we observed upon BiP depletion. To investigate this, an ATF6 reporter construct fused to a luciferase gene was used [31]. HEK293 cells were co-transfected with BiP shRNA and the ATF6 reporter construct and were subsequently analyzed for luciferase activity. Tunicamycin, a GlcNAc transferase inhibitor, which inhibits the glycoprotein synthesis, was used as a positive control for ATF6 activation [32]. As expected, BiP knockdown induced ATF6 release and reporter transcription (Figure 4A). In agreement with previous reports [30], Grp94, an ER chaperone in complex with BiP, was found to be upregulated upon BiP depletion (Figure 4B). Thus, the sensitization towards ricin might be induced by ER stress. In order to investigate this, cells were stressed with tunicamycin for 21 h before they were subjected to ricin treatment for 3 h and protein synthesis was measured. As shown in Figure 4C, no sensitization towards ricin was observed in the tunicamycin treated cells. This indicates that ER stress signaling does not necessarily sensitize the cells to ricin and is probably not the reason for increased ricin toxicity in BiP depleted cells. This is in agreement with our previous report showing that increased levels of misfolded proteins protect against ricin toxicity [12]. Interestingly, the level of BiP was significantly increased upon incubation with tunicamycin (Figure 4D and [33]). This may explain why treatment with tunicamycin does not sensitize the cells towards ricin. In conclusion, induction of ER stress is not sufficient to enhance ricin sensitization.

## 3. Discussion

The ER chaperone BiP has since its discovery been ascribed several functions and is today accepted as a master regulator of ER function [4]. BiP belongs to the heat shock protein 70 (Hsp70) family, and is highly dependent on a number of interacting partners, including co-chaperones, nucleotide exchange factors and signaling molecules [1]. BiP and its interaction partners have also been implicated in pathology of both infectious and inherited diseases as well as cancer [1]. In addition, bacterial toxins seem to utilize the function of BiP to exert their cytotoxic effect. Shiga toxin [17,18], cholera toxin [19] and the AB_5_ subtilase cytotoxin [34] all belong to the AB_5_ family of toxins and utilize BiP to perform their cytotoxic effects, either by hijacking its ERAD-related activity or by directly inactivating it. Although tested in a genome-wide RNAi screen and shown not to be required for intoxication, a further role of BiP in ricin translocation and toxicity has so far not been specifically addressed [15,16]. Bassik *et al.*, tested the sensitization to toxins in their shRNA screen, however BiP was not included in the pool [15].

In the present paper, we show that high levels of BiP reduce ricin toxin translocation to the cytosol with subsequent protection against cell cytotoxicity. In agreement with this, we found that the cells were sensitized towards ricin upon BiP depletion by shRNA treatment, and this seems to be due to increased toxin retrotranslocation to the cytosol. Depletion of BiP has previously been shown to induce the unfolded protein response and apoptosis [30], whereas in human U373 astroglioma cells BiP shRNA did not activate UPR [35], indicating that BiP might regulate these signaling transduction pathways differently depending on the cell lines. In our experiments, BiP depletion for up to three days induced the ATF6 signaling pathway and led to an increase in the Grp94 protein level. However, the ER stress inducer tunicamycin protected the cells against ricin and this result reveals that ER stress *per se* was not sufficient to increase ricin cytotoxicity. We have previously shown that an increase in misfolded polypeptides protects against ricin [12], a finding in agreement with the results reported here. In addition, tunicamycin significantly increased the expression of BiP, indicating that the lack of sensitization towards ricin could at least partly depend on the high level of BiP.

Pull down experiments showed that in the ricin A-chain pull down, BiP wt was present but not the substrate binding mutant. These data were obtained using cell lysates and it can therefore not be excluded that another protein or a complex is involved in the binding. Nevertheless, our findings suggest that BiP acts as a negative regulator of ricin toxicity through an interaction, which inhibits ricin translocation to the cytosol. This is in contrast to both cholera and Anthrax toxin, which seem to be dependent on BiP for their cytosolic access [19,22]. Thus, BiP might play different roles depending on the toxin. 

Ricin is today classified as a level B biothreat and antitoxin treatment protocols are under investigation [36]. Furthermore, vaccination protocols against ricin toxicity have been shown to give promising results using a non-toxic recombinant ricin molecule with mutations in its active sites [37,38]. On the other hand, ricin has been linked to antibody fragments or growth factors that bind specifically to target cells in order to create immunotoxins used in cancer treatment (reviewed in [39]). Thus, it is important to reveal the trafficking pathways exploited by ricin including the proteins involved in the different transport steps. In conclusion, a better understanding of ricin transport can help to develop efficient treatments both towards ricin intoxication and cancer. 

## 4. Experimental Section

### 4.1. Reagents and Antibodies

Ricin, HEPES, lactose, tunicamycin and digitonin were from Sigma Chemical Co. (St Louis, MO, USA). [^3^H]leucine and Na_2_^35^SO_4_ were purchased from GE Healthcare (Princeton, NJ, USA). The following antibodies were used: rabbit anti-ricin (Sigma Chemical Co., St Louis, MO, USA), mouse anti-BiP (BD Transduction Laboratories, San Diego, CA, USA), mouse anti-myc (Santa Cruz Biotechnology, Santa Cruz, CA, USA) mouse anti-His (Nordic Biosite AB, Täby, Sweden) and rat anti-Grp94 (Stressgen, Michigan, USA). HRP-coupled secondary antibodies were from GE healthcare. 

### 4.2. Cell Culture

Human Embryonic Kidney 293 (HEK293) cells were grown in DMEM (BioWhittaker, MD, USA) supplemented with 10% fetal calf serum (FCS), 100 U/mL penicillin and 100 U/mL streptomycin (Invitrogen, Carlsbad, CA, USA) with 5% CO_2_ in a 37 °C incubator. The wells were coated with Poly-L-lysine (Sigma Chemical Co., St Louis, MO, USA) according to the manufacturer’s protocol.

### 4.3. DNA Constructs, Transfection, Protein Expression and Pull down

The cDNA encoding human BiP WT and the point mutant P495L (located in the substrate binding site) [23] as well as the ATF6-luciferase reporter gene and the ATF6 negative control [40] were kindly provided by Prof. Ron Prywes (Columbia University, New York, NY, USA). The renilla reporter control, used in the luciferace experiments, was obtained from Promega Corporation (WI, USA). The short hairpin RNAs (shRNAs) against BiP were constructed using a shuttle vector containing a U6 promoter sequence [41]. The constructs were targeted against the sequences CCCGTCCAGAAAGTGTTGGG (shRNA1) and GAACCATCCCGTGGCATAG (shRNA2). The target specificity was tested using the NCBI EST database (http://www.ncbi.nlm.nih.gov/BLAST/). As a negative control, a U6 promoter fused to a degenerated hairpin was used. All constructs were sequenced. HEK293 cells were transiently transfected with FuGENE™ transfection reagent according to the manufacturer’s procedure (Boehringer Mannheim, IN, USA). The retroviral vector pLNCX2-EGFP [42] was included in the transfection protocols (10% of total DNA) to estimate the transfection efficiency and to visualize transfected cells.

Ricin A was amplified from pRA (a kind gift from Prof. Sjur Olsnes (The Norwegian Radium Hospital, Oslo, Norway), [24]) using the following primers: 5’-CATGCCATGGCAATATTCCCCAAACAATACCC-3’ and 5’-CCGCTCGAGAAACTGTGACGACGGTGG-3’. The amplicon was then cloned into pET21d (Novagen) using *Nco*I and *Xho*I. The first primer created an ATG and an *Nco*I site and the second primer introduced an *Xho*I site in frame with the vector-encoded 6XHis-tag. The resulting product was a ricin A-chain fused to 6xHis in its C-terminal end. Clones were sequenced and a positive construct was transformed into Rosetta bacteria (Novagen). Protein expression was performed using a standard procedure. Briefly, 250 mL of bacteria were grown at 37 °C until OD = 0.5 and then IPTG was added to a final concentration of 1 mM, and induction was continued for 3.5 h at 37 °C. Cells were then spun down and lysed in lysis buffer (50 mM Tris pH 8.0, 25 mM NaCl, 2 mM EDTA, lyzozyme and Complete Protease Inhibitors (Roche)) and left for 30 min at room temperature. Following this the lysate was sonicated for 5 min at 40% output, Triton X-100 was added to 1% final concentration, and the mix was left at 4 °C with constant rotation for 30 min. Finally, a 10 min centrifugation at 10,000 xg was performed and the cleared lysate was applied to an Ni-NTA column (Qiagen). Purification was then performed following the manufacturer’s instructions. The eluate was finally dialyzed overnight against water, protease inhibitors were added and the aliquoted solution was stocked at −20 °C.

HEK293 cells were transfected with the BiP constructs or an empty vector 24 h prior to cell lysis in the PD buffer (1% Triton X-100, 50 mM Hepes pH 7.5, 150 mM NaCl, 10% glycerol, supplemented with Complete Protease Inhibitors (Roche)) on ice. The lysates were centrifuged for 10 min at 10,000 ×g and the supernatants were pre-cleared with 50 μL Ni-NTA slurry per sample, for 15 min at room temperature. The lysates were then split in two, and half of the samples were mixed with 1 μg His-Ricin A. After 1h of rotation at room temperature, 50 μL Ni-NTA was added to all the samples and the samples were rotated for an additional 15 min. The Ni-NTA slurry was then washed twice with PD buffer containing 10 mM imidazole, and resuspended in reducing sample buffer. The samples were separated by SDS-PAGE, transferred to Immobilin PVDF Transfer Membrane (Millipore, Billerica, MA, USA) and analyzed by Western blot.

### 4.4. Ricin Toxicity and Measurement of Protein Synthesis

HEK293 cells were transfected with the indicated construct or an empty control vector. Three days post transfection cells were washed in leucine free medium and incubated with different concentrations of ricin for 1 or 3 h. Optionally, the cells were pre-incubated with inhibitors for 30 min before ricin was added. Concentrations of inhibitors are indicated in the figure legends. The cells were then incubated in leucine free medium supplemented with 1 µCi/mL [^3^H]leucine for 20 min at 37 °C. Proteins were then precipitated with 5% TCA, the precipitates were solubilized in 0.1 M KOH and finally the radioactivity was measured in a β-counter.

### 4.5. Sulfation of Ricin Sulf-1 and Permeabilisation of Cells

Ricin A-chain containing a sulfation site in the C-terminus was produced, purified and reconstituted with ricin B-chain to form ricin sulf-1 as previously described [24]. Cells were incubated with 0.2 mCi/mL Na_2_^35^SO_4_ in DMEM without sulfate for 3 h before approximately 500 ng/mL of ricin sulf-1 was added, and the incubation was continued for 3 h at 37 °C. The cells were then washed with a 0.1 M lactose solution at 37 °C to remove surface-bound ricin sulf-1, and once with ice-cold PBS before lysis (lysis buffer: 0.1 M NaCl, 10 mM Na_2_HPO_4_, 1 mM EDTA, 1% Triton X-100, supplemented with Complete Protease Inhibitors; pH 7.4). For permeabilisation, the cells were first washed once with room tempered PBS and incubated for 5 min at room temperature with KOAc buffer (115 mM CH_3_COOK, 25 mM Hepes, 2.5 mM MgCl_2_; pH 7.4) containing 3 μg/mL digitonin [12] followed by a 30 min incubation on ice to allow cytosolic proteins to diffuse into the medium. The supernatants were centrifuged to remove cell debris and nuclei for 10 min at 5000 rpm in an Eppendorf centrifuge. Sulfated ricin sulf-1 was immunoprecipitated from the supernatant with rabbit anti-ricin antibodies immobilized on protein A-Sepharose CL-4B (GE Healthcare, Princeton, NJ, USA). Finally, the beads were washed with ice-cold PBS supplemented with 0.35% Triton X-100, and the adsorbed material was analyzed by SDS-PAGE under reducing conditions. For the detection of ^35^SO_4_^2-^-labeled ricin, the proteins were transferred to a PVDF membrane. Membranes were exposed to Kodak BioMax MR films (Rochester, NY, USA) at room temperature. Signal intensities of the bands were quantified using ImageQuant 5.0 software (GE Healthcare, Princeton, NJ, USA). The radioactivity in the cell lysates was always measured to control that there were no differences in the total amount of isotope incorporated under the different conditions (data not shown). 

The efficiency of permeabilization and leakage of ER resident proteins were estimated as described [12]. 

### 4.6. ATF6 Activation Assay

HEK293 cells were transfected with 45% of the indicated constructs together with 45% of the firefly luciferase reporter construct and 10% of the *Renilla* luciferase reporter pRL-SV40P as an internal control for transfection efficiency. 3 days post transfection the cells were either left untreated or treated with 2 µg/mL tunicamycin for 8 h. Cells were then lysed and assayed for both firefly and *Renilla* luciferase activities using the Promega dual luciferase^®^ reporter assay (Promega, Sunnyvale, CA, USA) as described by the manufacturer. Luciferace activity was measured on a Victor2™ 1420 Multilabel counter (PerkinElmer). All experiments were performed with duplicate plates for each point.
